# Three-dimensional virtual modeling for lower limb deformity correction over intramedullary nail

**DOI:** 10.3389/fsurg.2026.1814038

**Published:** 2026-07-01

**Authors:** Michal Goetz, Yair Gortzak, Lior Yosef Shabtai, Ron Qual, Nadav Graif, Eran Golden, Solomon Dadia, Dror Ovadia, Roy Gigi

**Affiliations:** 1 Pediatric Orthopedic Department, Dana-Dwek Children's Hospital, Tel Aviv Sourasky Medical Center, Tel Aviv, Israel; 2 Tel Aviv University Faculty of Medical and Health Sciences, Tel Aviv-Yafo, Israel; 3 Department of Orthopedic Oncology, Tel-Aviv Sourasky Medical Center, Tel Aviv, Israel; 4 Pediatric Orthopedic Department, Long Island Jewish Medical Center, New Hyde Park, NY, United States; 5 Levin Center of Surgical Innovation and 3D Printing, Tel Aviv Sourasky Medical Center, Tel Aviv, Israel; 6 Department of Pediatric Orthopedic Surgery, Dana Dwek Children’s Hospital, Tel Aviv Sourasky Medical Center, Tel Aviv, Israel

**Keywords:** 3D surgical planning, deformity correction, intramedullary nail, lower limb, patient-specific cutting guides

## Abstract

**Purpose:**

The use of intramedullary nails (IMNs) for deformity correction and limb lengthening has become increasingly established. However, performing acute deformity correction over an IMN remains technically demanding. The purpose of this study was to describe the application of 3D virtual surgical planning and patient specific 3D-printed cutting guides for the management of complex lower-limb deformities in young patients.

**Methods:**

This retrospective, single-center study included 21 patients (mean age: 12 years) who underwent surgery using IMNs for complex lower-limb deformities correction between May 2019 and June 2024. Virtual 3D femoral and tibial models were created for preoperative planning and used to design patient-specific cutting guides for the surgical correction. All patients were evaluated for preoperative and postoperative anteroposterior and lateral deformity angles, time to osteotomy union, and complications.

**Results:**

Twenty-one patients underwent correction of 23 extremities. According to an *a priori* classification, an excellent correction (residual angulation ≤5° in both planes) was achieved in 19/23 extremities (83%) and an acceptable correction (6–10° residual angulation, within the recognized tolerance for the growing skeleton) in 4/23 extremities (17%), no extremity demonstrated residual deformity >10°. Among the 20 patients available for follow-up, the mean time to radiographic union was 8.5 weeks (range, 4.5–18 weeks), excluding the cases treated with a PRECICE lengthening nail for combined limb deformity correction and bone lengthening. No major intraoperative or early postoperative complications occurred, two extremities (8.7%) developed delayed union that resolved without intervention, one planned secondary procedure was performed (4.3%), and one patient (4.8%) was lost to follow-up.

**Conclusion:**

Three-dimensional virtual planning and patient-specific cutting guides represent effective adjuncts for correcting complex lower-limb deformities using intramedullary nails in pediatric patients. This approach enhances the accuracy of preoperative planning and facilitates more precise and controlled surgical execution.

## Introduction

Lower limb deformities encompass a broad spectrum of abnormalities affecting the hip, knee, ankle, and foot (1–4). These deformities may occur in the coronal, sagittal, axial, or oblique planes and often involve complex multiplanar components that challenge both diagnostic assessment and surgical correction (1, 3, 5). Historically, correction of lower limb deformities has primarily relied on external fixation and submuscular plating techniques. However, intramedullary nails (IMN) have gained increasing acceptance as an alternative method for deformity correction and limb lengthening (3–8). This shift has largely been driven by the recognized limitations of external fixation, including patient discomfort and the prolonged duration of treatment, which may contribute to increased patient frustration and reduced compliance (9–12). In addition, external fixation is associated with several well-documented complications, including muscle contracture, nerve injury occurring in approximately 9% of patients (13), and pin-site infections, with rates reported to be as high as 27.4% in systematic reviews (14, 15). Submuscular bridge plating represents an additional fixation strategy, particularly for metaphyseal and juxta-articular deformities (16, 17). However, plate fixation has several important limitations, including restricted postoperative weight-bearing, limited applicability for multiple osteotomies, and a potential increased risk of delayed union or nonunion (16, 17). In patients with congenital bone disorders such as Osteogenesis Imperfecta and Fibrous Dysplasia, intramedullary fixation is often preferred because of poor bone quality and the higher risk of implant failure with plate constructs (18, 19). Furthermore, unlike plate fixation, selected intramedullary lengthening nails allow simultaneous deformity correction and gradual limb lengthening (20, 21). Furthermore, intramedullary nails avoid prolonged immobilization and reduce complication rates (3). Patients experience fewer scars and improved comfort, as they are spared the irritation associated with pins and wires (16), and complications such as hardware infections or muscle-tethering contractures are uncommon with these devices (10). By permitting early joint motion without transfixing the skin and soft tissues, intramedullary nails also support quicker recovery and better postoperative range of motion compared with external fixators (17). The choice between external fixation, intramedullary nailing, and submuscular plating is therefore guided by deformity location, magnitude, the need for gradual correction or lengthening, and patient-specific factors. External fixation, including Ilizarov and hexapod systems, remains the standard for large multiplanar deformities requiring gradual correction, for cases with active or quiescent infection, and for patients with very small bone diameter in whom intramedullary access is not feasible (3, 4, 10). Intramedullary nailing is generally preferred for diaphyseal deformities in patients with adequate canal diameter, for those requiring simultaneous correction and limb lengthening with a motorized nail, and for fragile-bone conditions such as osteogenesis imperfecta where the rod also serves a prophylactic role (3, 6–8, 18, 19). Submuscular plating retains a defined role in metaphyseal and juxta-articular deformities where intramedullary fixation cannot reliably control the short juxta-articular segment (16, 22, 23).

Deformity correction using intramedullary nails, however, presents unique technical challenges, particularly in pediatric orthopedics when addressing complex multiplanar deformities and significant rotational abnormalities (24). These cases often require extensive osteotomies to allow proper nail placement and to achieve optimal correction. To overcome these potential obstacles, particularly in multi-planar deformities, an accurate and meticulous approach to preoperative planning and intraoperative execution is essential.

The utilization of 3D technologies for deformity correction is rapidly emerging in orthopedic surgery, enhancing both preoperative planning and intraoperative execution to improve overall surgical outcomes (25). It includes reconstructing virtual 3D models from CT scans and MRIs in order to better understand complex anatomy and to assist in preoperative osteotomy planning by considering multiple surgical approaches. Additionally, 3D printing of 1:1 scale anatomical models facilitates surgical simulation and preoperative planning by enabling accurate assessment of rotational deformity correction, determination of the required coronal and sagittal wedge dimensions, prediction of the final limb length, and selection of appropriate implant size (20, 21, 25–27). The patient-specific virtual anatomical model can subsequently be used to manufacture 3D-printed patient-specific instruments (PSIs), including surgical guides (jigs) designed to assist intraoperative execution (25, 28, 29).

3D printing technology has been used in pediatric orthopedic surgery, primarily with plate fixation, to assist with complex deformity corrections. Recent studies have explored its application with intramedullary nailing, but clinical evidence remains limited (12, 24, 25, 30). Unlike plate-based correction, where the implant is positioned along the bone surface and a cutting guide can be referenced directly to the future plate position, intramedullary nailing requires the implant to traverse a precisely reamed canal that must be coaxial with the corrected anatomical axis. Once the nail is inserted, no postoperative or intraoperative adjustment of alignment is possible, so the osteotomy geometry must be planned and executed with a degree of accuracy that is not typically demanded of plate-based corrections (3, 6). From a preoperative planning standpoint, accurate CT- and MRI-based three-dimensional reconstruction allows the surgeon to identify the true center of rotation of angulation (CORA), to quantify rotational malalignment, to predict the postoperative limb-length discrepancy after wedge closure, and to anticipate cortical impingement against a straight intramedullary implant (12, 25, 27).

The aim of this retrospective case series was to describe the application of a standardized 3D virtual planning and patient-specific cutting-guide workflow for the correction of complex lower limb deformities using intramedullary nail fixation, and to evaluate its clinical and radiographic outcomes, including correction accuracy, time to union, and complication rates. We hypothesized that integrating three-dimensional virtual planning with patient-specific instrumentation would enable accurate and reproducible multiplanar deformity correction using intramedullary nail fixation while minimizing complications.

## Methods

This retrospective case series was conducted at the Limb Deformity Service of the Department of Pediatric Orthopaedic Surgery, Tel Aviv Sourasky Medical Center, a tertiary referral center specializing in the management of congenital, developmental, metabolic, and post-traumatic lower limb deformities in pediatric and young adult patients. Institutional review board approval was obtained prior to study initiation, and the requirement for informed consent was waived due to the retrospective design of the study. Ethical approval was granted by the Institutional Review Board of Tel Aviv Sourasky Medical Center (approval number: TLV-0641-19).

All consecutive patients who underwent deformity correction using three-dimensional (3D) virtual surgical planning, patient-specific 3D-printed cutting guides, and intramedullary nail fixation between May 2019 and June 2024 were retrospectively reviewed. Inclusion criteria consisted of: (1) complex femoral or tibial deformities requiring correction with intramedullary fixation (2) utilization of CT-based three-dimensional virtual planning and patient-specific instrumentation as part of the surgical workflow (3) deformities requiring complex osteotomy planning, including wedge osteotomies, in conjunction with intramedullary nail fixation and (4) availability of complete clinical and radiographic follow-up data. Patients with congenital, metabolic, developmental, and post-traumatic deformities were included. Exclusion criteria included patients treated without intramedullary fixation, cases in which patient-specific instrumentation was not utilized, insufficient imaging or follow-up data, and patients managed exclusively with external fixation or plate fixation techniques.

A total of 21 patients met the inclusion criteria and were enrolled in the study (Table 1). Demographic, clinical, radiographic, and operative data were collected from institutional medical records and imaging archives. The following parameters were collected: (1) patient demographics, including age and sex at the time of surgery (2) deformity characteristics, including etiology, anatomical location, and preoperative deformity measurements on anteroposterior and lateral radiographs(3) operative details, including the number and level of osteotomies performed and the type of intramedullary implant utilized and (4) postoperative outcome measures, including postoperative radiographic alignment, time to radiographic union, duration of follow-up, and perioperative or postoperative complications.

**Table 1:** Patient demographics, underlying diagnoses, and clinical deformity patterns at the time of surgery (*n* = 21 patients, 23 operated extremities).

Case	Age (y)	Sex	Bone	Side	Underlying pathology	Clinical deformity
1	13 + 3	M	Femur	L	Post-traumatic	Femoral procurvatum + varus
2	8 + 0	M	Tibia	L	CPT due to NF1	Tibial varus + procurvatum
3	13 + 5	M	Tibia	R	Pathologic fracture over osteofibrous dysplasia	Proximal tibial valgus + distal tibial varus (double deformity)
4	17 + 9	F	Tibia	L	OI type I with anterior cortex nonunion	Tibial procurvatum
5	14 + 11	F	Femur	L	Enchondromatosis (Ollier disease)	Distal femoral valgus + 5 cm LLD
6	12	F	Femur	R	McCune–Albright syndrome with pathological fracture over a nail	Proximal femoral varus
7	13 + 3	F	Tibia	R	OI type I	Midshaft tibial valgus + procurvatum
8	10 + 5	M	Tibia	R	CPT due to NF1	Midshaft tibial valgus + procurvatum
9	13 + 1	M	Tibia	L	Fibrous dysplasia with tibial pathological fractures	Midshaft tibial valgus + procurvatum
10	4 + 6	M	Tibia	R	OI type III	Midshaft tibial procurvatum
11	15 + 8	M	Femur	L	Enchondromatosis (Ollier disease)	Femoral valgus + procurvatum + external rotation + LLD
12	15 + 3	M	Femur	R	Enchondromatosis (Ollier disease)	Femoral varus + 6.5 cm LLD
13	9 + 1	M	Tibia	R	Osteofibrous dysplasia	Tibial valgus
14	16 + 5	F	Femur	R	Maffucci syndrome	Femoral varus + recurvatum
15	31	M	Tibia	L	Post-traumatic	Tibial varus + recurvatum
16	8 + 3	M	Femur	L	Fibrous dysplasia	Femoral valgus from pathological fracture fixated with IMN
17	15 + 7	F	Tibia	L	OI type IV	Tibial valgus + procurvatum
18	11 + 9	M	Femur	L	OI type I	Femoral varus + procurvatum
19a	4	F	Tibia	R	OI type XIV	Tibial procurvatum
19b	4	F	Tibia	L	OI type XIV	Tibial varus + procurvatum
20	8 + 1	M	Femur	R	OI type IV	Femoral varus
21a	2 + 2	M	Femur	R	OI type III	Femoral varus + procurvatum
21b	2 + 2	M	Tibia	R	OI type III	Tibial varus + procurvatum

Age in years and months (e.g., 13 + 3 = 13 y 3 mo). M, male; F, female; L, left; R, right; LLD, limb-length discrepancy; CPT, congenital pseudarthrosis of the tibia; NF1, neurofibromatosis type I; OI, osteogenesis imperfecta; IMN, intramedullary nail; Patients 19 and 21 underwent simultaneous bilateral procedures (suffixes a and b denote each operated extremity).

Etiology was stratified into congenital/developmental/metabolic deformities and post-traumatic deformities because these groups differ substantially in bone biology, skeletal maturity, deformity complexity, implant selection, and expected healing characteristics. Anatomical deformity location (femur vs. tibia) and deformity pattern were additionally evaluated because these factors may influence surgical planning, osteotomy configuration, fixation strategy, and postoperative alignment outcomes.

All radiographic measurements were performed using the digital measurement tools integrated within the institutional radiographic software by a fellowship-trained pediatric orthopedic surgeon with more than five years of experience in limb deformity correction. Angular measurements were performed on anteroposterior and lateral radiographs of the involved bone segment. Because deformity correction was performed over an intramedullary nail, which restores alignment along the anatomical axis of the corrected bone, the deformity was quantified using the anatomical axis method as described by Paley (31). For each affected segment, the proximal and distal anatomical axes of the bone were drawn on the radiograph, their intersection defined the CORA, and the angle formed between these two axes at the CORA represented the magnitude of the deformity. Coronal-plane deformity (varus or valgus) was measured on the anteroposterior radiograph, and sagittal-plane deformity (procurvatum or recurvatum) was measured on the lateral radiograph, using the same axis-based technique. The preoperative deformity angle was compared with the postoperative angle measured along the corrected anatomical axis at last follow-up, and the residual deformity was defined as the residual angulation between the proximal and distal anatomical axes after correction. All measurements were performed using a PACS-integrated digital measurement software program previously validated for pediatric orthopedic radiographic parameters, which demonstrated high intra- and inter-observer reliability across the relevant lower-limb angles (32). Radiographic union was defined as bridging callus formation with continuity across at least three cortices on follow-up radiographs. Correction outcomes were classified *a priori* according to the residual deformity at last follow-up, based on accepted thresholds for clinically and biomechanically tolerable malalignment in pediatric long-bone deformity correction (31, 33). Excellent correction was defined as residual angulation ≤5° in both the coronal and sagittal planes, acceptable correction as residual angulation of 6–10° in either plane, a range within the recognized tolerance for the growing skeleton, where remodeling potential and the absence of clinically meaningful functional impairment have been documented in pediatric long-bone deformity correction, and incomplete correction as residual angulation >10° in either plane.

Postoperative complications were prospectively recorded throughout the follow-up period and graded according to the Clavien–Dindo classification adapted for orthopedic surgery (34), in which Grade I represents any deviation from the normal postoperative course not requiring pharmacologic or surgical intervention, Grade II requires pharmacologic treatment, Grade IIIa requires intervention without general anesthesia, Grade IIIb requires intervention under general anesthesia, Grade IV is a life-threatening complication, and Grade V is death. Delayed union was defined *a priori* as failure to achieve bridging callus across at least three cortices by 6 months postoperatively, and nonunion as the absence of progressive radiographic healing for at least three consecutive months beyond 9 months postoperatively. Loss to follow-up was defined as the absence of clinical or radiographic evaluation within the institutional system after the index procedure, regardless of cause.

### Statistical analysis

Given the descriptive nature of this retrospective case series, statistical analyses were limited to summary measures. Continuous variables are reported as mean ± standard deviation with range, and as median where appropriate; categorical variables are reported as frequencies and percentages. Pre- and postoperative coronal and sagittal angular measurements were compared using the Wilcoxon signed-rank test, selected because of the small sample size and the non-normal distribution of deformity magnitudes confirmed by the Shapiro–Wilk test. A two-sided *p* value < 0.05 was considered statistically significant. No imputation was performed for missing data, and extremities with incomplete radiographic data were excluded from the corresponding analyses (denominators reported throughout). Analyses were performed using IBM SPSS Statistics, version 27.0 (IBM Corp., Armonk, NY, USA).

### Study workflow and 3D planning technique

**Figure 1:**
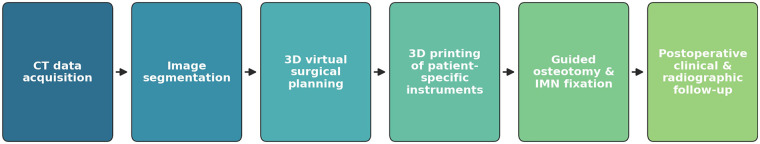
Study workflow showing the sequential stages of the integrated three-dimensional virtual planning and patient-specific instrumentation workflow: CT acquisition, segmentation and three-dimensional reconstruction, virtual deformity analysis and osteotomy planning, three-dimensional printing of anatomical models and patient-specific cutting guides, intraoperative correction and intramedullary nail fixation, and postoperative clinical and radiographic follow-up. CT, computed tomography; IMN, intramedullary nail.

The study workflow (Figure 1) comprised six stages: (1) CT data acquisition; (2) image segmentation; (3) three-dimensional virtual surgical planning; (4) 3D printing of anatomical models and patient-specific instruments (PSIs) (5) operative correction using guided osteotomies and intramedullary nail fixation assisted by the 3D-printed PSIs and (6) postoperative clinical and radiographic follow-up.

### Segmentation

Preoperative CT imaging of the lower extremities was obtained using a SOMATOM X.cite scanner (Siemens Healthineers, Erlangen, Germany). To minimize radiation exposure in this pediatric population, CT acquisition was limited to thin-cut 1-mm slices of the anatomical segment undergoing correction (femur or tibia), rather than the entire lower extremity. This targeted imaging protocol was based on the principles of deformity correction using intramedullary nail fixation, in which correction is performed according to the anatomical axis of the involved bone segment. In contrast to plate fixation or external fixation techniques, which may permit postoperative or intraoperative adjustment of alignment, intramedullary nailing relies on precise restoration of alignment between a straight implant and the corrected bone segment. Therefore, imaging of the involved segment alone was considered sufficient for accurate preoperative planning while adhering to radiation reduction principles.

The two-dimensional CT datasets were imported into FDA-approved image-processing software (Mimics, Materialise NV, Leuven, Belgium; or Intellispace Portal versions 9 and 11, Philips Healthcare, Best, The Netherlands), segmented, and reconstructed into patient-specific three-dimensional digital bone models. Following segmentation, the models were exported as STL files and imported into dedicated FDA-approved computer-aided design (CAD) software (3-matic, Materialise NV) for surgical planning and guide design (25).

### Virtual planning of corrective osteotomy: the mirroring and “Shish-Kebab” techniques

At our institution, a dedicated in-house 3D Medical Innovation Laboratory provides advanced services including CT- and MRI-based three-dimensional anatomical reconstruction, computer-assisted surgical planning, and 3D printing of patient-specific instruments. The laboratory is capable of manufacturing patient-specific cutting guides, anatomical models, and customized surgical tools designed specifically for each procedure (35).

The initial and, in our opinion, most critical stage of the workflow was a collaborative preoperative planning session involving the orthopedic surgeon and biomedical engineer, during which the deformity characteristics, osteotomy strategy, implant trajectory, and overall correction plan were established.

**Figure 2:**
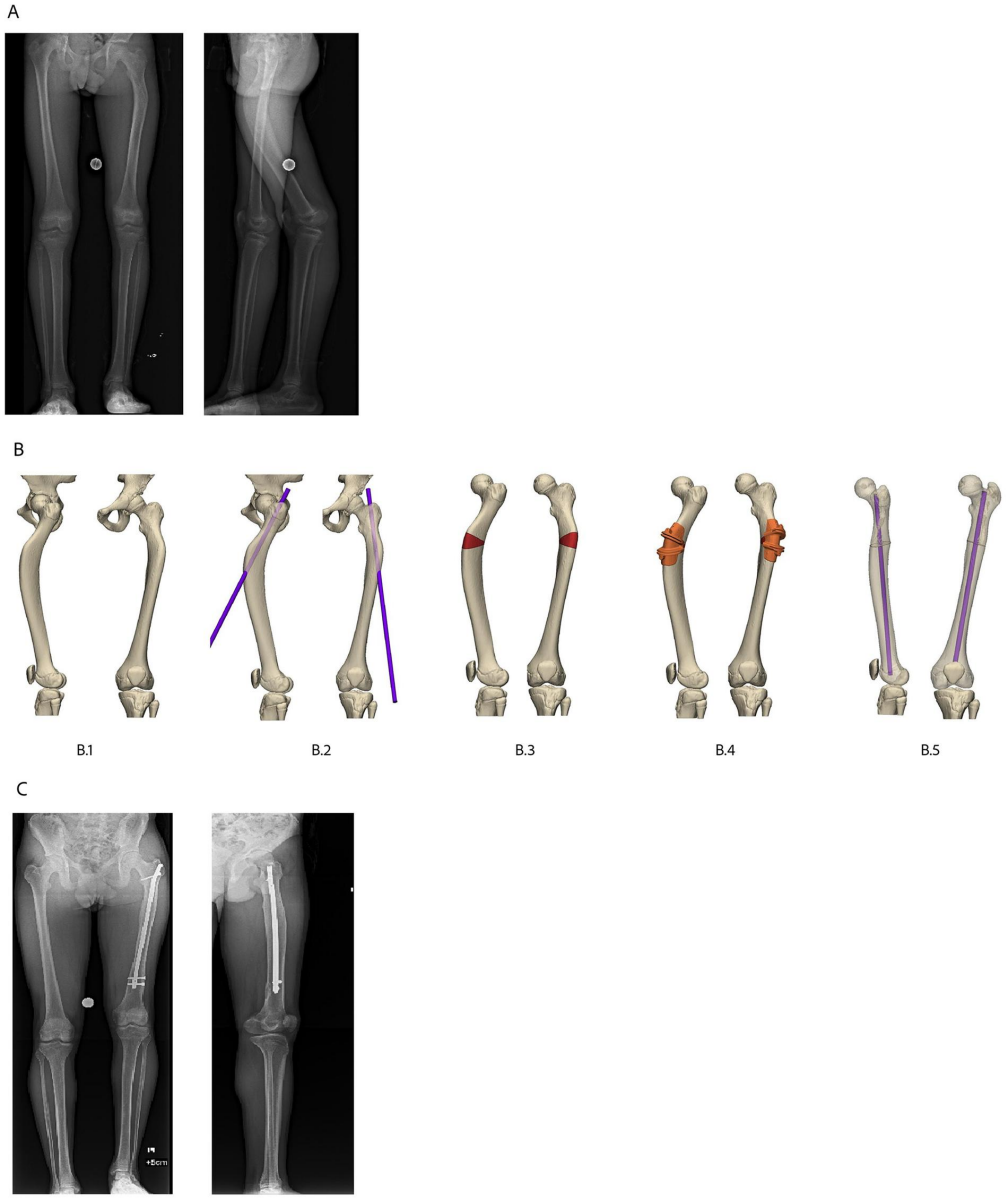
Representative case of mirror-based virtual deformity correction in a 13-year-old boy with a post-traumatic three-plane femoral deformity and limb-length discrepancy. **(A)** Preoperative radiographs. **(B)** Virtual planning sequence, including mirrored reconstruction, simulated intramedullary nail insertion, wedge-osteotomy planning, patient-specific guide design, and final corrected alignment. **(C)** Seven-month postoperative radiographs demonstrating restored alignment and radiographic union. IMN, intramedullary nail.

For patients with unilateral deformities, corrective osteotomies were planned using a virtual mirroring technique (Figure 2) (25, 35). Following segmentation, the unaffected contralateral bone model was mirrored and superimposed onto the affected bone using CAD software aligned to either the proximal or distal segment. The deformed bone segment was subsequently manipulated to achieve alignment with the mirrored reference model while maintaining fixation of the opposite segment. The primary osteotomy plane was positioned as close as possible to the CORA (center of rotation of angulation) and oriented perpendicular to the principal deformity plane. A secondary osteotomy plane was then automatically generated to create a closing wedge osteotomy. The resulting wedge geometry incorporated correction of the coronal, sagittal, and axial deformity components.

**Figure 3:**
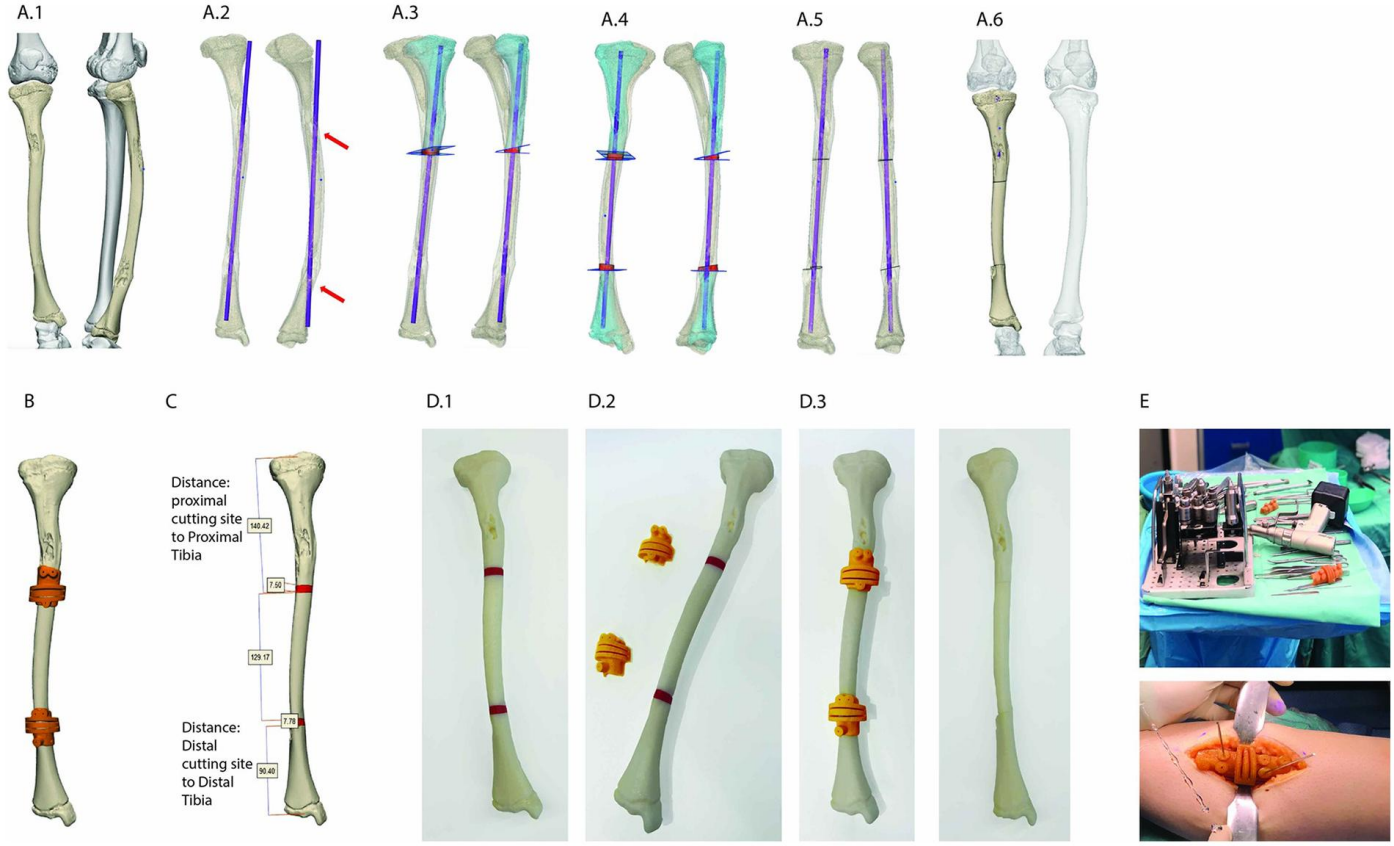
Representative case of three-dimensional “Shish-Kebab” planning in a 13-year-old boy with tibial deformity secondary to osteofibrous dysplasia nonunion. **(A)** Stepwise virtual planning with simulated nail trajectory, wedge generation, osteotomy planning, and final corrected alignment. **(B)** Patient-specific cutting guide design. **(C)** Planned guide positioning using joint-reference measurements. **(D)** Three-dimensional printed model used for surgical simulation before and after wedge removal. **(E)** Intraoperative application of the sterilized cutting guide. IMN, intramedullary nail.

For patients with bilateral deformities, a virtual “Shish-Kebab” planning technique was utilized, (Figure 3) based on the principles originally described by Sofield and Millar (36). Following segmentation, a virtual intramedullary nail was inserted through the deformed bone model. Whenever cortical impingement was encountered, additional osteotomy planes were created to permit unobstructed passage of the implant. Once one extremity was virtually corrected, the mirroring technique was subsequently applied to facilitate planning of the contralateral side.

Rotational alignment was assessed and corrected during the virtual planning phase. Using the CT-based 3D model, the optimal rotational correction required to restore anatomical alignment following coronal and sagittal correction was determined. The planned rotational correction was measured directly on the virtual model. Intraoperatively, rotational alignment was reproduced either with patient-specific cutting jigs incorporating proximal and distal Kirschner wire reference sleeves or by independent placement of proximal and distal Kirschner wires according to the predefined rotational correction parameters.

Upon approval of the surgical plan, patient-specific cutting guides and anatomical bone models were manufactured using 3D printing technology through an in-house, hospital-integrated workflow utilizing FDA-approved CAD platforms (Mimics and 3-matic, Materialise NV). The cutting guides were printed on a Fortus 450mc (Stratasys) using biocompatible, high-strength, heat-resistant ULTEM™ 1010 resin at a resolution of 330 μm. Following production, the PSIs underwent washing, double packaging, and sterilization using a standard 135 °C autoclave cycle.

### Surgical technique

All procedures were performed with the patient in the supine position.

#### Step 1—nail entry and initial preparation

Standard intramedullary nail entry points were utilized according to implant orientation, including the greater trochanter for antegrade femoral nails, the distal femur for retrograde femoral nails, and the proximal tibia for tibial nails.

#### Step 2—osteotomy according to the virtual surgical plan

The proximal and distal joint orientation lines were identified relative to the deformity. The patient-specific cutting guide was positioned according to predefined measurements from the adjacent joint landmarks. Following skin incision and layered dissection, the guide was seated directly onto the exposed bone surface. Osteotomies were performed using an oscillating saw through the predefined guide slots, and wedge completion was achieved using an osteotome. In selected cases, temporary stabilization using monolateral external fixation pins was applied during canal preparation and reaming.

#### Step 3—preparation of the medullary canal and nail fixation

Canal reaming was performed through the designated entry point in either an antegrade or retrograde direction. In selected complex deformities, reaming was initiated directly through the osteotomy site and extended proximally and distally, a technique that provided improved control during canal preparation.

Reaming facilitated creation of a uniform intramedullary canal, enabled insertion of the largest permissible implant diameter, and reduced intramedullary pressure prior to insertion of the definitive implant, thereby decreasing the risk of iatrogenic fracture.

Following canal preparation, the intramedullary nail was inserted. Proximal and distal interlocking screws were utilized only in cases involving rigid trauma-style intramedullary nails. The cutting guides were subsequently removed, and final alignment and implant positioning were verified fluoroscopically.

## Results

Twenty-one patients underwent corrective surgery on a total of 23 lower-limb segments using the described technique (Table 1). Two patients (cases 19 and 21) underwent simultaneous bilateral procedures. Fourteen of 21 patients were male (67%) and seven of 21 were female (33%). The mean age at surgery was 12.3 ± 6.1 years (median 13.1 years, range, 2.2–31.0 years). Twenty of 21 patients (95%) were skeletally immature at the time of surgery (mean age 11.3 ± 4.4 years), whereas one adult patient with a post-traumatic deformity was included according to the predefined inclusion criteria. The femur was the treated segment in 10/23 extremities (44%) and the tibia in 13/23 extremities (57%). Right- and left-sided procedures were similarly distributed (52% vs. 48%, respectively).

Because deformity etiology may influence implant selection, correction strategy, and healing potential, patients were stratified according to underlying diagnosis. Most patients had congenital, developmental, or metabolic bone disorders (19/21, 91%), whereas two patients (9.5%) presented with post-traumatic malunions. Osteogenesis imperfecta was the most common diagnosis (8/21, 38%), followed by enchondromatosis (Ollier disease) (3/21, 14%), fibrous dysplasia (2/21, 9.5%), osteofibrous dysplasia (2/21, 9.5%), congenital pseudarthrosis of the tibia associated with neurofibromatosis type I (2/21, 9.5%), McCune–Albright syndrome (1/21, 4.8%), and Maffucci syndrome (1/21, 4.8%). All three patients with Ollier disease required management of associated limb-length discrepancy. Two patients (cases 5 and 12) underwent simultaneous deformity correction and gradual limb lengthening using a PRECICE intramedullary lengthening nail. Case 11 underwent staged treatment, consisting of initial deformity correction and fixation with an antegrade trauma intramedullary nail followed by subsequent exchange to a PRECICE lengthening nail for gradual limb lengthening. Deformity characteristics and anatomical location are summarized in Table 1. Multiplanar deformities involving both the coronal and sagittal planes were observed in 13/23 extremities (57%), whereas isolated single-plane deformities were present in 10/23 extremities (44%). Clinically significant limb-length discrepancy was present in 3/23 extremities (13%). Tibial deformities more commonly demonstrated multiplanar involvement and associated limb-length discrepancy, whereas femoral deformities were more frequently isolated angular deformities, although no statistically significant difference between femoral and tibial deformities was identified because of the limited cohort size. The mean preoperative coronal deformity was 21.6 ± 12.3° (range, 5–64°; *n* = 20 extremities), and the mean preoperative sagittal deformity was 29.2 ± 17.3° (range, 4–75°; *n* = 17 extremities).

The mean clinical and radiographic follow-up duration for the remaining cohort was 22.0 ± 13.3 months (median 19.5 months, range, 4–53 months). Sixteen patients (16/21; 76%) had completed a minimum of 12 months of follow-up at the time of submission.

**Figure 4:**
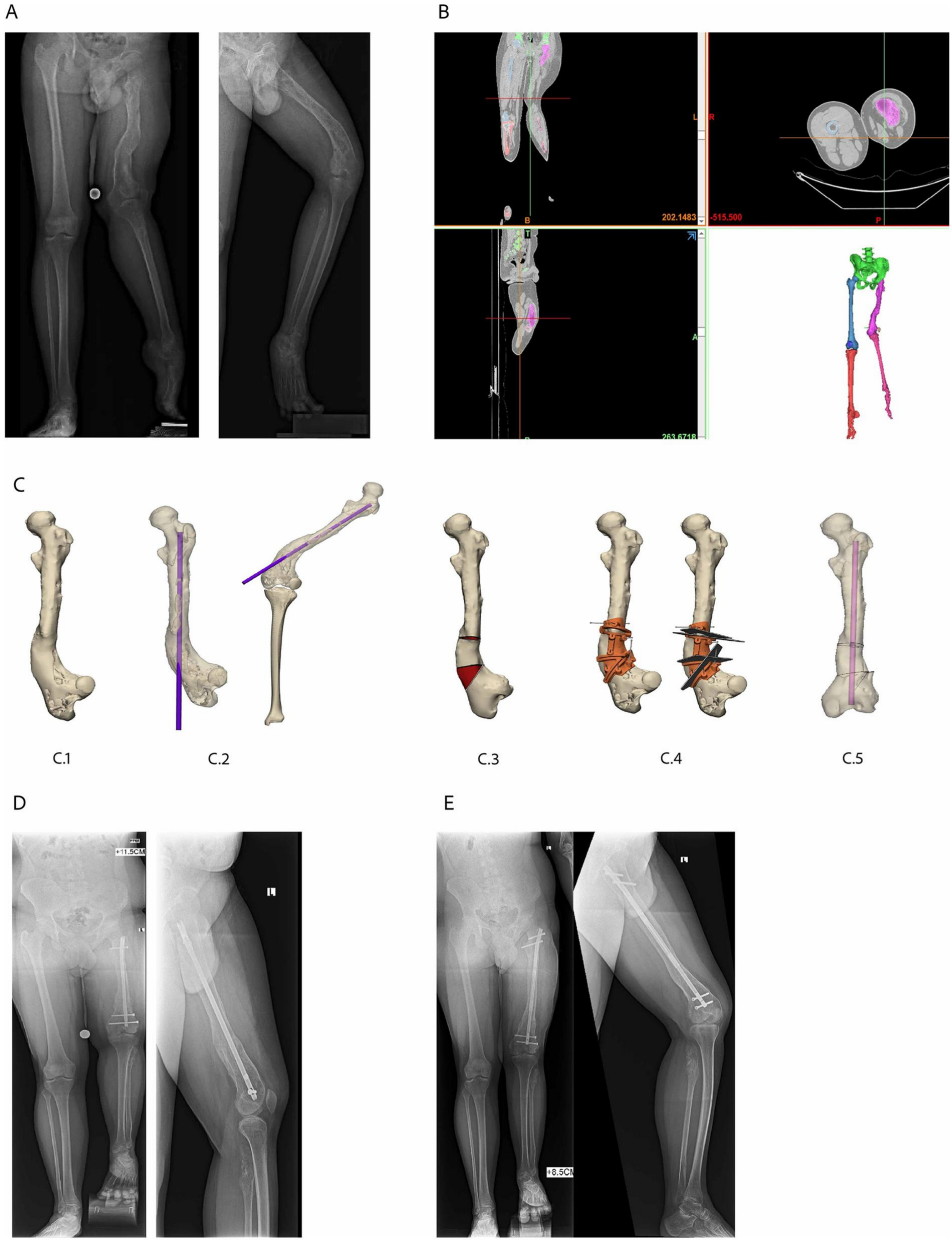
Representative complex case (case 11) of femoral deformity correction in a 16-year-old boy with enchondromatosis (ollier disease). **(A)** Preoperative radiographs. **(B)** CT-based three-dimensional reconstruction demonstrating multiplanar deformity. **(C)** Virtual planning sequence, including deformity analysis, wedge-osteotomy planning, patient-specific guide design, and corrected alignment. **(D)** Radiographs demonstrating consolidation following deformity correction and trauma intramedullary nail fixation. **(E)** Follow-up radiographs after exchange to a PRECICE lengthening nail, demonstrating maintained alignment and complete consolidation. CT, computed tomography; IMN, intramedullary nail.

Postoperative radiographic findings at last follow-up are summarized in Table 2 and illustrated in the representative clinical cases shown in Figure 4. Substantial multiplanar correction was achieved in all 23 extremities, with no case demonstrating residual deformity greater than 10° in either the coronal or sagittal plane. The mean residual coronal deformity decreased from 21.6 ± 12.3° preoperatively to 1.5 ± 3.0° postoperatively (Wilcoxon signed-rank, *p* < 0.001), corresponding to a mean coronal correction of 20.0 ± 10.5° (*n* = 20). The mean residual sagittal deformity decreased from 29.2 ± 17.3° to 0.9 ± 2.3° (Wilcoxon signed-rank, *p* < 0.001), corresponding to a mean sagittal correction of 28.0 ± 16.3° (*n* = 17). According to the *a priori* classification, an excellent correction (residual angulation ≤5° in both planes) was achieved in 19/23 extremities (83%), residual coronal angulation ≤5° was obtained in 20/23 extremities (87%) and residual sagittal angulation ≤5° in 21/23 extremities (91%). An acceptable correction (residual angulation 6–10° in either plane) was observed in 4/23 extremities (17%), comprising the following residual deformities at last follow-up: case 2 (7° varus), case 9 (7° valgus with 5.5° procurvatum), case 21a (10° varus with 2° procurvatum), and case 21b (5° varus with 9° procurvatum). No case met the predefined criterion for incomplete correction (residual angulation >10° in either plane). The two extremities with the largest residual deformities (cases 21a and 21b) belonged to a young patient with osteogenesis imperfecta who underwent simultaneous ipsilateral femoral and tibial correction, the residual 10° varus and 9° procurvatum fall within the range considered biomechanically and functionally acceptable for the pediatric long bone, where ongoing growth and skeletal remodeling are expected to mitigate residual angulation without compromising mechanical-axis restoration or function (31, 33). No patient demonstrated symptomatic malalignment, gait disturbance attributable to residual deformity, or required revision surgery for malalignment during the follow-up period.

**Table 2:** Surgical details, radiographic outcomes, complications, and clinical follow-up for the 23 operated extremities (21 patients).

Case	Pre-op deformity	Pre-op AP (°)	Pre-op Lateral (°)	Osteotomies (n)	Nail type	Post-op AP (°)	Post-op Lateral (°)	Complications	Follow-up (mo)
1	Femoral procurvatum + varus	30° varus	43° procurvatum	1	Antegrade trauma IMN	0°	0°	None	37
2	Tibial varus + procurvatum	28° varus	19° procurvatum	1	Fassier–Duval IMN + circular ex-fix	7° varus	0°	None	53
3	Proximal tibial valgus + distal tibial varus	Prox 24° valgus; Dist 10° varus	31° procurvatum	2	SLIM IMN + circular ex-fix	0°	0°	None	46
4	Tibial procurvatum	0°	24° procurvatum	1	Antegrade trauma IMN	0°	0°	Lost to follow-up	1
5	Distal femoral valgus + 5 cm LLD	25° valgus	0°	1	PRECICE lengthening nail	0°	0°	Delayed lateral cortex union (Clavien–Dindo I)	19
6	Proximal femoral varus	16° varus	0°	1	Antegrade trauma IMN	0°	0°	None	45
7	Midshaft tibial valgus + procurvatum	18° valgus	12° procurvatum	2	Fassier–Duval IMN + locking plate	0°	1° procurvatum	Locking plate removal for delayed cortex union (Clavien–Dindo IIIb)	31
8	Midshaft tibial valgus + procurvatum	19° valgus	18° procurvatum	1	Fassier–Duval IMN + circular ex-fix	0°	4° procurvatum	None	22
9	Midshaft tibial valgus + procurvatum	21° valgus	21° procurvatum	1	Antegrade trauma IMN	7° valgus	5.5° procurvatum	None	28
10	Midshaft tibial procurvatum	0°	43° procurvatum	2	SLIM IMN + circular cast	0°	0°	None	24
11	Femoral valgus + procurvatum + ER + LLD	35° valgus + 40° ER	45° procurvatum	2	Antegrade trauma IMN → PRECICE	0°	0°	None	24
12	Femoral varus + 6.5 cm LLD	12° varus	0°	1	PRECICE lengthening nail	0°	0°	None	20
13	Tibial valgus	21° valgus	0°	2	Fassier–Duval IMN + circular ex-fix	0°	0°	None	20
14	Femoral varus + recurvatum	15° varus	8° recurvatum	1	Antegrade trauma IMN	0°	0°	None	8
15	Tibial varus + recurvatum	13° varus	4° recurvatum	1	Antegrade trauma IMN	0°	0°	None	4
16	Femoral valgus from pathological fracture	19° valgus	0°	1	Antegrade trauma IMN	0°	0°	None	18
17	Tibial valgus + procurvatum	14° valgus	40° procurvatum	1	Fassier–Duval IMN	0°	0°	None	8
18	Femoral varus + procurvatum	10° varus	32° procurvatum	1	Fassier–Duval IMN	0°	0°	None	8
19a	Tibial procurvatum	0°	20° procurvatum	1	Fassier–Duval IMN	0°	0°	None	14
19b	Tibial varus + procurvatum	5° varus	22° procurvatum	1	Fassier–Duval IMN	0°	0°	None	14
20	Femoral varus	19° varus	0°	1	Fassier–Duval IMN	4.5° varus	0°	None	12
21a	Femoral varus + procurvatum	64° varus	40° procurvatum	1	SLIM IMN	10° varus	2° procurvatum	None	14
21b	Tibial varus + procurvatum	25° valgus	75° procurvatum	1	SLIM IMN	5° varus	9° procurvatum	None	14

AP, anteroposterior; ER, external rotation; ex-fix, external fixator; IMN, intramedullary nail; LLD, limb-length discrepancy; mo, months. Pre- and post-operative angular measurements were obtained on anteroposterior and lateral radiographs of the affected segment, measured between the proximal and distal anatomical axes at the centre of rotation of angulation; 0° indicates restoration of anatomical alignment in that plane. Complications are graded according to the Clavien-Dindo classification adapted for orthopaedic surgery. The arrow (→) in case 11 denotes a planned staged exchange of the intramedullary implant.

One patient (1/21; 4.8%; case 4 in Table 2) was lost to follow-up after relocating outside the institutional referral region shortly after surgery. No early postoperative complications were observed, and immediate postoperative radiographs demonstrated satisfactory correction. As correction was confirmed postoperatively, this patient was included in the alignment analysis but excluded from time-to-union and long-term outcome assessments.

Implant selection was individualized according to patient age, skeletal maturity, bone quality, canal diameter, deformity characteristics, and the need for simultaneous limb lengthening. Four implant systems were utilized across the 23 extremities: Fassier-Duval telescopic nails in 9/23 extremities (39%), rigid antegrade trauma nails in 7/23 (30%), SLIM nails in 4/23 (17%), and PRECICE motorized lengthening nails in 3/23 (13%). Telescopic Fassier-Duval nails were primarily selected for skeletally immature patients with osteogenesis imperfecta or fragile bone disorders to accommodate continued longitudinal growth while reducing the risk of peri-implant fracture. Rigid trauma-style nails were preferred in older patients with sufficient canal diameter and cortical bone stock requiring increased axial and rotational stability. SLIM nails were utilized in very young children with narrow medullary canals and severe deformity patterns. PRECICE nails were reserved for patients requiring simultaneous deformity correction and gradual limb lengthening.

Complications were graded according to the Clavien-Dindo classification adapted for orthopedic surgery. No intraoperative complications occurred, and there were no neurovascular injuries, deep or superficial infections, implant failures, compartment syndromes, thromboembolic events, fat embolism, or iatrogenic fractures during the follow-up period. Postoperative adverse events were recorded as follows. Two extremities (2/23, 8.7%) developed delayed cortical union without loss of alignment or progression to nonunion (case 5, treated with a PRECICE lengthening nail, and case 7, treated with an adjunctive locking plate construct); both were managed expectantly and were classified as Clavien–Dindo Grade I, as no pharmacologic or surgical intervention beyond routine follow-up was required to achieve consolidation. One extremity (case 7:1/23, 4.3%) subsequently underwent a planned secondary procedure under general anesthesia for removal of the adjunctive locking plate after radiographic consolidation, this reoperation is explicitly acknowledged as a Clavien-Dindo Grade IIIb event. No patient required revision surgery for malalignment, nonunion, infection, or implant failure, and no Grade IV or V complications occurred.

## Discussion

Intramedullary nails are increasingly utilized for limb deformity correction, owing to their lower complication rates and superior patient comfort. Benefits such as improved soft-tissue protection, rapid bone formation, and reliable consolidation contribute to early full weight-bearing without assistive devices (37–39). Advances in implant design including enhanced interlocking options and improved rotational control have further strengthened their role in cases where traditional treatment methods were limited, making intramedullary nails a desirable option for selected deformity patterns (40). However, deformity correction over an intramedullary device remains technically demanding, requiring precise preoperative planning, accurate CORA identification, and meticulous intraoperative execution, as repeatedly highlighted in the literature (3, 6, 9, 10, 41). In this context, our study explores how virtual computed 3D planning and patient-specific guides may support surgeons in addressing these challenges, particularly in complex multiplanar deformities, where no postoperative adjustments are possible and a precise three-dimensional alignment must be restored in a single stage.

In our series, careful virtual planning enabled safe and efficient correction for 21 patients with complex lower limb deformities that underwent virtual preoperative planning followed by deformity correction guided by intraoperative 3D-printed osteotomy jigs, with no intraoperative complications and a low rate of postoperative adverse events. We presented one of the most demanding cases of a 16-year-old boy with Ollier disease, who presented with a three-plane deformity of the left femur and a significant limb length discrepancy (Figure 4). Virtual 3D modeling revealed a more precise CORA and additional multiplanar deformity components that were obscured on standard radiographs. By defining the true deformity and the eccentric intramedullary canal pathway, the model helped prevent malrotation and unintended translation. These findings underscore that conventional imaging alone may be insufficient, even in cases that appear straightforward. For these reasons, we consider virtual surgical planning as an important component of preoperative preparation and an effective means of optimizing clinical outcomes.

Over time, multiple strategies have been developed for deformity correction and simultaneous bone lengthening with IMNs. The reverse planning method (RPM), introduced by Baumgart (9), begins by defining the ideal final alignment and then working backward to the existing deformity using preoperative templates. Although elegant and effective, RPM is best suited for relatively simple deformities and does not fully address complex three-dimensional corrections requiring precise multiplanar wedge osteotomies (42). Fixator-assisted nailing offers another option, in which an external fixator is temporarily applied to obtain alignment before stabilizing the bone with an intramedullary nail (43, 44). However, this technique still relies on accurate preoperative planning and has limited capability for achieving true multiplanar correction, particularly when deformities span both the coronal and sagittal planes. Similarly, blocking (Poller) screws have been used to guide reaming and maintain alignment during IM nailing. Dabash et al. further refined this concept with the reverse-rule-of-thumbs (RROT) guideline (41). Yet, even with these refinements, the technique requires meticulous preoperative planning and remains constrained in complex multiplanar deformities. Additionally, in young patients with narrow canals, placing blocking screws is technically challenging and increases the risk of cortical breach or iatrogenic fracture, further limiting the practicality of this approach in the population most commonly presenting with such deformities.

The transition from traditional surgical methods to the integration of 3D technologies has progressively reshaped modern medicine as a whole, with a particularly notable impact on surgical disciplines. Across multiple medical fields, 3D virtual modeling, simulation tools, and patient-specific guides have been shown to enhance preoperative planning, deepen anatomical understanding, and improve intraoperative precision (45–51). These technologies allow surgeons to rehearse complex procedures, anticipate anatomical variations, and tailor surgical strategies to the individual patient benefits that have been documented in areas ranging from orthopedics and maxillofacial surgery to neurosurgery and cardiovascular interventions. This paradigm shift reflects a broader evolution in surgical practice, where personalized, image-based workflows increasingly support accuracy, safety, and efficiency.

In orthopedic surgery, several groups have explored virtual planning and patient-specific instruments as an advancement beyond traditional planning methods. Victor and Premanathan (52) demonstrated early use of CT-based 3D models and printed guides for precise corrective osteotomies, followed by plate fixation. A related concept was described by Chai et al. (53), who combined virtual osteotomy planning with a customized intramedullary nail. More recently, Oba et al. (54) applied CT-based navigation and a 3D model to simulate the nail pathway and osteotomy planes in a complex tibial deformity. Collectively, these studies show growing interest in 3D planning and patient-specific guides, yet clinical applications involving intramedullary nails remain limited.

Building on the limitations of existing methods and the emerging evidence supporting virtual planning, our workflow offers the additional key advantage of reliable assessment and correction of rotational deformities, particularly in cases with complex multiplanar abnormalities that can be difficult to evaluate clinically. The 3D planning process enables precise quantification of rotational malalignment and allows surgeons to predetermine the exact correction required before entering the operating room. By translating the virtual plan into surgery through patient-specific jigs or aligned K-wire reference points, this approach may enhance the accuracy of rotational correction compared with conventional methods.

While the applicability of patient-specific cutting jigs in very young children with OI or CPT is limited due to small bone diameter, fragile cortices, and the risk of intraoperative fractures even with Kirschner wire insertion, the 3D virtual planning process itself provides substantial advantages. In this population, standard radiographs are frequently suboptimal because of difficulty standing or maintaining consistent positioning, limiting accurate assessment of complex deformities. Virtual 3D models allows precise visualization of alignment, osteotomy planes, and rotational components. Preoperative virtual planning enables surgeons to rehearse the procedure, anticipate obstacles, and optimize implant selection, including simulating canal size,which may reduce intraoperative uncertainty and technical errors. Further studies are required to determine which components of this technology are beneficial in young patients and whether they translate into improved clinical or radiographic outcomes. Additionally, in cases requiring multi-level correction, as compared to a classical Sofield procedure (36) in which the entire shaft of the bone was exposed, we used limited skin incisions after establishing the location of the deformity and through using surgical cutting guides (jigs). This reduces periosteal damage, blood loss, and infection (55). While the traditional “shish-kebab” technique remains reliable in curved bone surgery, complex multi-apical deformities present challenges that may be difficult to anticipate intraoperatively. “Virtual rehearsal” using computed 3D models helps visualize deformity geometry, canal eccentricity, and rotational alignment in advance, adding precision and safety, especially in atypical anatomy or multi-osteotomy cases. The Virtual 3D planning primarily serves as an adjunct to the surgeon's existing technique rather than a procedure requiring its own learning curve. By allowing surgeons to practice on an accurate model before surgery, it may shorten any surgery learning process and enhance operative precision.

This study is limited by a relatively short follow-up period, especially among the youngest patients, for whom long-term remodeling, recurrence, and implant-related issues may emerge later. However, the present work focuses on the planning and execution advantages provided by 3D modeling rather than on long-term outcomes. Larger studies with matched controls and extended follow-up are needed to determine whether these technological enhancements translate into durable clinical and radiographic benefits. The retrospective, single-center, single-arm design without a control group of patients treated with conventional planning is an inherent limitation. As such, this study cannot establish whether the observed alignment and complication outcomes are causally attributable to the 3D virtual planning workflow rather than to surgeon experience, case selection, or other unmeasured factors. The clinical question of whether 3D virtual planning and patient-specific instrumentation improve outcomes over conventional planning will require a prospective comparative study, ideally with matched or randomized controls. The heterogeneity of the cohort (multiple diagnoses, implant types, and anatomical segments) further limits subgroup analysis but reflects the real-world referral pattern of complex pediatric deformities at a tertiary center. An additional limitation is the absence of validated patient-reported outcome measures (PROMs). These instruments were not routinely collected during the study period, and outcome assessment was therefore restricted to clinical examination, radiographic alignment, time to union, and complication rates. We also acknowledge a 4.8% loss-to-follow-up rate (one patient) related to geographic relocation, which may introduce a degree of attrition bias even though the absolute number affected is small.

When considering 3D printing methodologies, there are several drawbacks to consider. First, Radiation exposure is an important consideration in pediatric imaging. In our workflow, CT acquisition is limited to the specific segment requiring correction rather than the entire limb, thereby minimizing radiation in accordance with ALARA (“as low as reasonably achievable”) principles. Current low-dose pediatric extremity CT protocols, including 1-mm slice thickness and iterative reconstruction. Second, The additional time required for preoperative planning with an engineer and medical designer, together with the cost of patient-specific instrumentation (approximately $1,500–$2,000), may not yet be fully justified in all settings. Nevertheless, costs can be significantly reduced when the planning workflow is integrated within the hospital service, as in our institution, and the setup process becomes streamlined. Further research is needed to evaluate the cost-effectiveness of this technology, particularly in its potential to reduce complications, reoperations, and operative time. Last, the use of cutting jigs typically requires larger soft tissue exposure to ensure a precise fit against the anatomical surface. While this theoretically diminishes the minimally invasive nature of the implant, standard techniques such as blocking screws (56) are not always feasible in complex cases or small bones. Moreover, our surgical technique for complex cases involves creating the intramedullary canal in a retrograde fashion, working distally and proximally from the osteotomy site, which already necessitates a larger incision. In our opinion, this approach provides superior control during canal preparation and significantly reducing the risk of translation or iatrogenic fractures.

## Conclusion

In this case series, integrating CT-based 3D virtual planning and patient-specific cutting guides into intramedullary nail correction of complex multiplanar lower limb deformities was feasible and was associated with satisfactory short-term radiographic alignment and a low complication rate. The workflow may be a useful adjunct to established techniques in selected pediatric and adolescent patients with complex deformities, particularly when multiplanar geometry is difficult to appreciate on plain radiographs.

## Data Availability

The raw data supporting the conclusions of this article will be made available by the authors, without undue reservation.
